# Effectiveness of Hyperbaric Oxygen Therapy in SARS-CoV-2 Pneumonia: The Primary Results of a Randomised Clinical Trial

**DOI:** 10.3390/jcm12010008

**Published:** 2022-12-20

**Authors:** Jacek Siewiera, Klaudia Brodaczewska, Natalia Jermakow, Arkadiusz Lubas, Krzysztof Kłos, Aleksandra Majewska, Jacek Kot

**Affiliations:** 1 Department of Hyperbaric Medicine, Military Institute of Medicine—National Research Institute, 04-141 Warsaw, Poland; 2 Laboratory of Molecular Oncology and Innovative Therapies, Military Institute of Medicine—National Research Institute, 04-141 Warsaw, Poland; 3 Department of Internal Diseases Nephrology and Dialysis, Military Institute of Medicine—National Research Institute, 04-141 Warsaw, Poland; 4 Department of Infectious Diseases and Allergology, Military Institute of Medicine—National Research Institute, 04-141 Warsaw, Poland; 5 Postgraduate School of Molecular Medicine (SMM), Warsaw Medical University, 02-091 Warsaw, Poland; 6 National Centre for Hyperbaric Medicine, Institute of Maritime and Tropical Medicine, Medical University of Gdansk, 81-519 Gdynia, Poland

**Keywords:** coronavirus disease-19 (COVID-19), hyperbaric oxygen therapy (HBOT)

## Abstract

Mortality in COVID-19 is mainly associated with respiratory failure, cytokine storm, and macrophage activation. Oxygenation and anti-inflammatory effects of Hyperbaric Oxygen Therapy (HBOT) suggest that it is a promising adjunct treatment for COVID-19. Repeated sessions of HBO with standard COVID-19 therapy were used to reduce the inflammation and increase oxygenation. We evaluated the safety and efficacy of HBOT in avoiding the replacement ventilation and/or ECMO and its effect on the inflammatory process. Twenty-eight moderate-to-severe COVID-19 patients were randomized into control or HBOT group. HBOT patients participated in 5 hyperbaric sessions (60 min). Before and after each session blood gas levels and vital parameters were monitored. Blood samples were collected for extended biochemical tests, blood morphology and immunological assays. There were 3 deaths in the control, no deaths in the HBOT group. No adverse events leading to discontinuation of HBOT were observed and patients receiving HBOT required lower oxygen delivery. We observed decrease in CRP, ferritin and LDH and increase in CD3 in HBOT group compared to control. This study confirmed the feasibility and safety of HBOT in patients with COVID-19 and indicated HBOT can lead to alleviation of inflammation and partial restoration of T cell responses.

## 1. Introduction

The Severe acute respiratory syndrome coronavirus 2 (SARS-CoV-2) leads to a disease termed coronavirus disease-19 (COVID-19). The first cases of COVID-19 were identified in 2019 in China. The SARS-CoV-2 virus became known as highly infectious, spread between persons by droplet transmission. The disease rapidly spread throughout China, with its epicentre in Wuhan [1] and on 11 March 2020 the World Health Organization (WHO) announce that COVID-19 had reached pandemic status [2]. As of 1 December 2022, globally, there have been 645,008,998 confirmed cases of COVID-19 and 6,640,432 fatalities, with a total of 6,354,298 confirmed cases and 118,340 fatalities reported in Poland at the time of the writing the present study [3].

Currently, there are lots of publications on the pathophysiology of COVID-19. From the clinical point of view, the pathomechanisms of the cellular effects of the virus resulting in cytokine release are important [4,5]. The main hypothesis supported by many reasonable publications is the so-called cytokine storm, which is actually a severe inflammation with massive macrophage activation [6,7,8].

As a viral infection, COVID-19 is characterized by type Th1 protective immune response [9], with contribution of virus-specific CD8+ cytotoxic T cells (Tc) and CD4+ helper (Th) lymphocytes [10], and memory T cells maintained after infection. Cytotoxic T cells are observed in blood from day 7 after symptom onset [11] and their high levels are required for virus clearance; however, their activity can be compromised during SARS-CoV-2 infection compared to other viral diseases [12]. T helper (Th) cells, primarily as a source of proinflammatory cytokines like IFN-γ, TNF-α, or IL-2, are important mediators of effective response against SARS-CoV-2. Disturbed CD4+ responses can contribute to ineffective immunity and severe disease outcomes [13]. Patients with symptomatic COVID-19 often present with lymphopenia, and lymphocyte counts (total, CD4+ and/or CD8+) are proposed predictive factors for disease severity. Therefore, adjunctive therapies restoring T cell responses, without exacerbating inflammation, can be beneficial in SARS-CoV-2 infected patients.

Viral coagulopathy, which results in pulmonary embolism; venous, arterial, and capillary thrombosis; lung endothelial injury; and the accompanying pulmonary complications of pulmonary embolism, is the second form of the negative viral effects on humans. The exact mechanisms underlying this process are still unclear and require further investigation. Mortality in COVID-19 is mainly associated with acute, severe and progressive respiratory failure termed Acute Respiratory Distress Syndrome (ARDS). Early mortality rates for ARDS in COVID-19 patients in Wuhan Province, China, exceeded 90%. Most clinicians note the rapid progression of localised lung lesions leading to fibrosis. High levels of IL-6 [14] and the lack of significant efficacy of antiviral treatment point to an inflammatory aetiology.

Severe hypoxia leading to patient death is what all hypotheses have in common. In fact, reversal of hypoxia is an essential part of COVID-19 therapy [15,16]. It is logical that all oxygenation methods including normobaric oxygen therapy, artificial ventilation, extracorporeal oxygenation methods and, ultimately, hyperbaric oxygen therapy have been investigated for potential efficacy in the treatment of COVID-19 [17].

Hyperbaric Oxygen Therapy (HBOT) uses elevated environmental pressure (over 101.3 kPa = 1 ATA = 760 mmHg) to treat illness or injuries. In clinical practice, exposure pressure of 2.4–2.8 ATA lasting from 60 to 120 min is used, at which the arterial blood oxygen pressure is between 1800 mmHg and 2200 mmHg. During the patient’s exposure to hyperbaric conditions, 100% oxygen is usually used for breathing as it reaches all body tissues via the microcirculation, exerting therapeutic effects. Oxygen is a powerful trigger for cellular reactions [18]. The exact mechanism is difficult to be elucidated due to a non-specific action of oxygen on all cells. Only recently the review of 137 articles out of 9618 found in the PubMed was published [19]. Generally, HBO exerts the anti-inflammatory effect via decreasing of C-reactive protein, increasing anti-inflammatory interleukins, and decreasing levels of the pro-inflammatory cytokines interferon-γ (IFN-γ), nuclear factor kappa B (NF-κB) and TNF-α. Additionally HBO induces pro-angiogenetic response and stimulates stem cell production [20,21,22,23]. Dose-reaction relation is still to be established, especially when comparing hyperoxia between normobaric and hyperbaric conditions [24].

The broad and physiological anti-inflammatory effects of HBO supported by extensive experimental and clinical evidence suggest that HBO is a promising treatment for COVID-19-associated ARDS [25,26,27]. Opinions of different authors strongly emphasising the rationale for the use of HBOT in COVID-19, even in the absence of sufficient clinical evidence yet, have been and are being published [28,29,30,31,32,33,34,35,36,37].

Until the launch of this study, there was no prospective randomised controlled trial (RCT) on the use of HBOT in COVID-19. Apart from a single published clinical case study [38], and reasonable research hypotheses based on previous primary study results in indications other than COVID-19 [39], there have been no strong studies evaluating the efficacy of HBOT in COVID-19. The interest in the possibility of using HBOT in COVID-19 in other research centres is high, as evidenced by the phase 2 trials registered in the ClinicalTrial.gov database (NCT04327505 and NCT04332081) and attempts to use this therapeutic modality in different countries as salvage therapy even in the absence of clinical trials (authors’ data).

Only a few cases of HBOT use in severe cases of COVID-19 respiratory failure could be found in the literature [38,40]. All of them concerned patients treated in a hospital in Wuhan (China), but have received international commentaries [41]. All of the 5 patients who received 3 to 8 sessions of HBOT at 24-h intervals showed clinical improvement and improved arterial blood gas parameters. Unfortunately, due to the small sample size, these results can only be classified as low-level evidence according to the classification of scientific reports (single case series without control group).

The research hypothesis of the project assumed that the use of repeated sessions of HBO as an adjunct therapy to standard COVID-19 therapy in moderate-to-severe patients can reduce the systemic and local inflammatory response in the lung tissue and pulmonary microcirculation and more rapidly extinguish the so-called cytokine storm [6,42]. Patients with increasing respiratory failure requiring oxygen therapy and possible need for ventilator therapy were eligible. Randomisation was performed after patient qualification for the study.

We evaluated the safety and efficacy of HBOT in avoiding the need for replacement ventilation and/or ECMO and the rate of resolution of the inflammatory process as measured by inflammatory cell and inflammatory cytokine levels compared to a control group without HBOT.

## 2. Materials and Methods

### 2.1. Study Design and Participants

**Figure 1:**
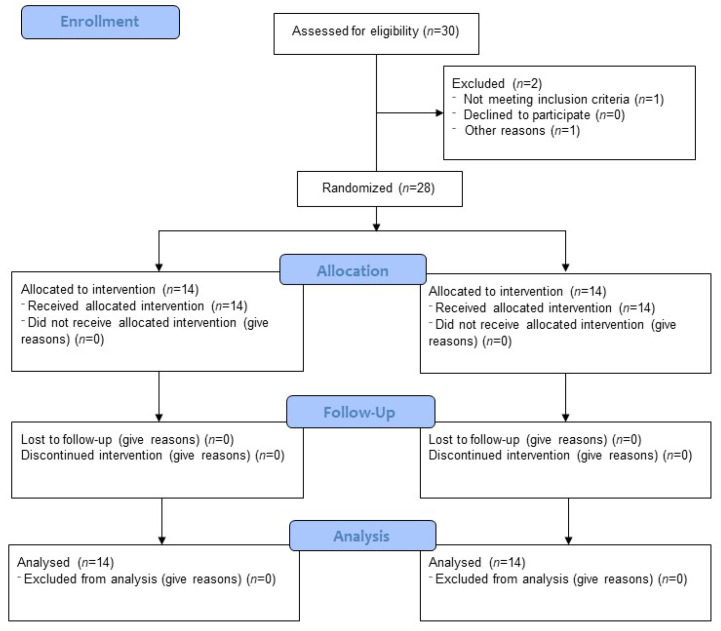
Consort flow diagram.

A randomized control trial was planned to be conducted as a prospective interventional study comparing standard treatment (Control Group, C) with Hyperbaric Oxygen Therapy (HBOT) added as the adjunctive procedure to the standard treatment for COVID-19 (HBOT Group). The protocol was approved by the Ethical Committee, registered at EudraCT (2020-002722-90) and funded by the Polish Medical Research Agency (grant 2020/ABM/COVID19/0043). According to the power analysis we planned to recruit 60 patients over 1 year. After recruitment of 30 patients, who completed the screening phase and signed consent for participation in the clinical trial, the study was terminated due to inability to recruit more patients (as allowed by the protocol) and subjected to partial analysis. One patient was excluded due to instant improvement after steroid medication (ceased to meet the inclusion criteria). In the second case, exclusion criteria were identified before treatment onset. Finally, 28 patients completed the protocol. Figure 1 shows the participant flow through the trial.

**Figure 2:**
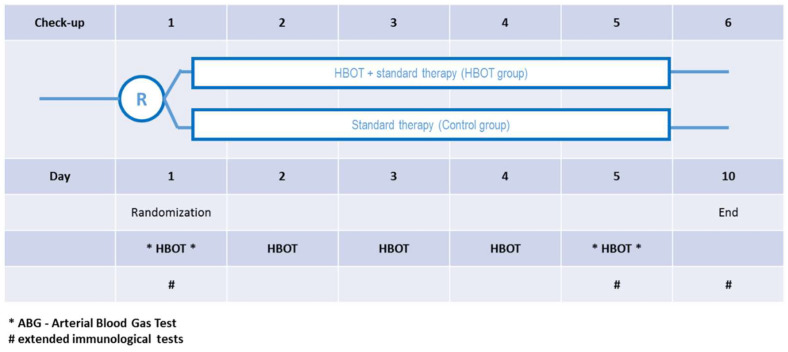
Protocol of Randomised Clinical Trial of Hyperbaric Oxygen Therapy in SARS-CoV-2 pneumonia.

The protocol is presented on the Figure 2. Briefly, after recruitment each patient was randomised either to the C group or to the HBOT group. In both groups, the standard treatment was the same. In the HBOT group patients participated in 5 hyperbaric sessions in the multiplace walk-in hyperbaric chamber breathing 60 min of 100% oxygen under pressure of 2.5 ATA using face masks. The total time of session was about 80 min, which also includes time of compression and decompression. Each patient was attended by the qualified medical personnel. Before and after each session blood gas analysis was conducted and vital parameters recorded, including oxygen dose NEWS score. Additionally, blood samples were taken for extended biochemical tests, blood morphology and immunological assays, as described later.

### 2.2. Blood Serum Collection

For each blood collection, peripheral blood was drawn into a serum tube (Vacutainer SST™ II Advance, BD, Warsaw, Poland) and allowed to clot for 30 min at room temperature (RT). To obtain serum, blood samples were fractionated by centrifugation at 2000× *g* for 15 min at RT, then supernatants were aliquoted, frozen, and stored at −80 °C.

### 2.3. Interleukin 6 Detection in the Serum

Enzyme-linked immunosorbent assay (DuoSet ELISA, R&D System, Minneapolis, MN, USA) was used to measure IL-6 levels in serum samples. The assay was performed according to the manufacturer’s protocol. Briefly, serum samples were thawed on ice, centrifuged at 2500× *g* for 15 min at 4 °C, and 100 µL of sample was tested in duplicates on primary antibody coated and blocked 96 well plates. Detection was performed with the use of HRP substrate, TMB. Plate spectrophotometric reader (VarioScan Lux, ThermoFisher Scientific, Waltham, MA, USA) at 450 nm was used to measure optical density. Standard curve for recombinant IL-6 and blanked absorbance measurements were performed.

### 2.4. Immunofluorescence Staining of Peripheral Blood Cells

Peripheral blood samples were collected in EDTA-anticoagulated tube (Blood Collection tube Vacutainer^®^, BD, Warsaw, Poland). Whole blood was used for extracellular markers immunofluorescence staining, 100 µL of blood was stained with anti-CD4-APC (# IM2468, Beckman Coulter, Brea, CA, USA), anti-CD8-APC-AF700 (# B49181, Beckman Coulter Brea, CA, USA), anti-CD3-APC-AF750 (# A94680, Beckman Coulter, Brea, CA, USA) and anti-CD45-KrO (# B36294, Beckman Coulter, Brea, CA, USA) for 30 min at RT. Unstained blood was used as negative control for each patient, while single stainings were used for compensation settings and gating strategy. After staining, erythrocytes were lysed for 15 min at RT using BD FACSTM Lysing Solution (BD Bioscience, Franklin Lakes, NJ, USA) and washed twice in PBS. Data were analysed by flow cytometry using CYTOFLEX software v.2.3.0.84 (Beckman Coulter, Brea, CA, USA). Debris was gated out on FCS/SSC dot plot and 30,000 events in “Cells” gate were acquired. Then, CD45+ cells were gated, followed by CD3+. Within the lymphocyte gate, the percentage of CD4+ and CD8+ cells was indicated.

### 2.5. Statistics

The analysis was performed using the R program (version 4.1.2.) with the rstatix library (Chi^2^ test, Friedman test, Mann-Whitney U test and Wilcoxon test). The scheme of analysis included testing for within-group effects before and after the applied therapy—10 days after the launch of the study, as well as between-group comparisons of the first and last measurement. A temporal analysis from day 1 to day 5 of the study was performed only in the case of arterial blood gas levels. Data gaps in the final measurement were filled by the Last observation carried forward (LOCF) method. Due to lack of agreement with normal distribution in almost all results by group (Shapiro-Wilk test *p* < 0.05), non-parametric tests were used in the analyses. Intra-group analyses were performed using the Wilcoxon test, and inter-group analyses were conducted using the Mann-Whitney test. Bonferroni correction was applied in both tests. The analysis of arterial blood gas results for within-group (measurement) and between-group (HBOT vs. C group) effects was performed using the Friedman test.

## 3. Results

### 3.1. Participants

Thirty patients of the Military Medical Institute, including 24 males and 6 females, aged 24–78 years (mean age 55 ± 13.4), hospitalised for SARS-CoV-2 infection from 1 March 2021 to 3 February 2022, participated in the study. All subjects signed a consent to participate in the study. Two patients were excluded due to the failure to meet the inclusion criteria. In the control group, 1 patient refused to continue participation on day 10, and 1 patient, a woman aged 51 years, refused to participate in the study on day 1. Following randomisation, 14 patients were assigned to the HBOT group, and 14 patients were placed in the Control group (C). In most cases, the HBOT started within 24 h from randomization, which was on average 5 days after the initial onset of symptoms. There were 3 deaths in the control (C) group, and no deaths in the HBOT group. The difference in death rate did not reach statistical significance (21.4% vs. 0.0%, *p* = 0.067). No adverse events (AEs) leading to discontinuation of any single HBOT sessions were observed in the HBOT group (0% of 65 HBOT sessions, 95%CI: 0–6%). Twenty-seven patients had comorbidities. The most common diagnoses were hypertension and insulin-dependent diabetes mellitus. There was no statistically significant difference in the prevalence of comorbidities between the C and the HBOT group (0/14 HBOT vs. 2/14 C; Chi^2^ = 0; *p* > 0.999). In 5 patients (HBOT: *n* = 4; C: *n* = 1), COVID-19 viral pneumonia was the only diagnosis. Most patients (*n* = 22, 73.3%) had several comorbidities (including one excluded). No statistically significant difference was observed between groups C and HBOT in age (56.07 ± 14.02 vs. 52.08 ± 13.51, Mann-Whitney U test, U = 83.5, *p* = 0.52), or sex (2/14 F vs. 4/14 F, Chi^2^ = 0; *p* > 0.99). Patient status according to the National Early Warning Score (NEWS) was similar in both groups at baseline (HBOT: M = 2.35, SD = 1.3; C: M = 2.5, SD = 1.1; *p* = 0.964) and final HBOT measurement: M = 1.72, SD = 0.99; C: M = 2.36, SD = 1.8; *p* = 0.962). As part of their treatment, all patients received subcutaneous anticoagulants and corticosteroids. Twenty-seven patients (including 2 patients excluded from the study) received antibiotic therapy, 5 patients were treated with remdesivir (HBOT: *n* = 3; C: *n* = 2) and one patient was treated with tocilizumab (in the HBOT group).

### 3.2. Blood Gas Test

The degree of lung injury was determined, among other things, using the Horowitz factor (PF, PaO2/FiO2), and the severity of normobaric oxygen therapy was measured by determining the amount of oxygen used. One of the therapeutic goals was to maintain normal oxygenation as measured by blood saturation (SpO2) levels. In fact, this was achieved in all patients. Statistical analysis showed that mean SpO2 and PiO2/FiO2 values did not differ significantly between groups throughout the study (Table 1). An analysis with the Friedman test showed no differences between the groups in successive measurements in both saturation levels (SpO2 (%): F = 1.2; *p* = 0.878), PiO2/FiO2: F = 6.4; *p* = 0.171). Temporal analysis within groups also showed no significant differences in SpO2 (HBOT: *p* = 0.398, C: *p* = 0.782), while for the Horowitz coefficient in the HBOT group, the temporal effect was at trend level (*p* = 0.068), in group C it was not significant (*p* = 0.835). The change in parameters over time is shown in Figure 2.

### 3.3. Oxygen Supply

**Figure 3:**
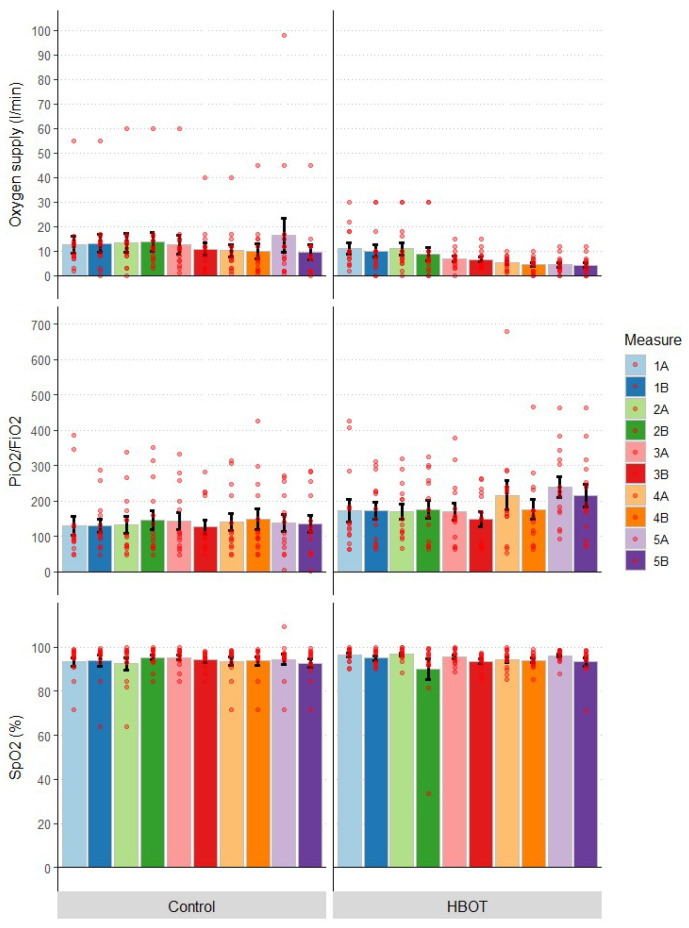
Change in blood gas levels and oxygen supply during the study by group; numbers indicate the day of measurement; A and B indicate measurement before or after the HBOT session on a given day, respectively; bars represent mean, error bars represent standard error, red dots indicate individual patient results; HBOT *n* = 14, Control *n* = 14.

Oxygen supply was tailored to individual patient needs to avoid hypoxemic events with SpO2 < 92%. In 6 patients, no passive oxygen therapy was used at different stages of the study, 13 patients used an oxygen reservoir mask during all or part of the measurements, while 2 patients breathed through a simple mask, one patient was administered oxygen via a mask with an oxygen reservoir and high-flow intranasal oxygen therapy (HFNOT) and one patient used a mask and oxygen sniffers, 6 patients used intranasal cannulas, and 1 patient required the use of a ventilator (in group C, the patient died on day 27 of hospital stay). Comparing the mean oxygen delivery throughout the study, a significantly lower oxygen delivery can be observed during treatment in the HBOT group compared to the control group (Figure 3). The analysis of intergroup differences in all measurements combined showed that the HBOT group had a lower oxygen supply throughout the study than the control group (U = 15459; *p* < 0.00001). In contrast, the intragroup differences in oxygen delivery by measurement were not statistically significant (day 1–5 before or after HBOT session), but on the second day after the session (U = 141; *p* = 0.050) and on day 5 before the session (U = 139; *p* = 0.061) the values were at the border of significance or at the trend level.

### 3.4. Biochemistry

**Figure 4:**
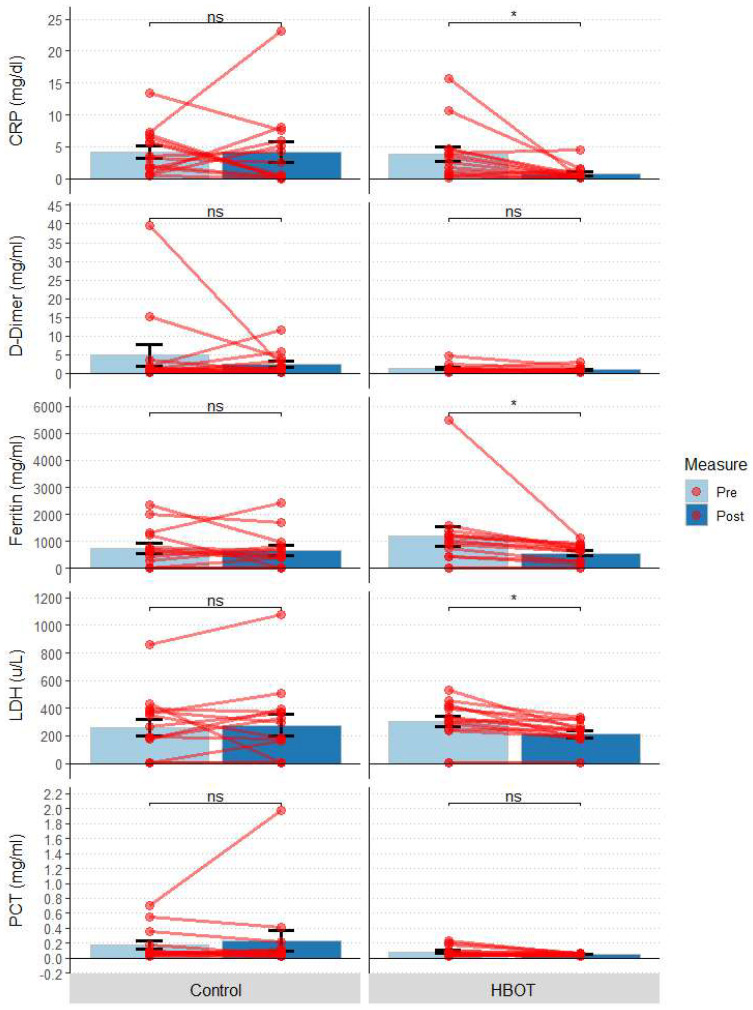
Biochemical parameters at the first measurement (Pre) and 10 days after study initiation (Post); bars represent mean, error bars represent standard error, red lines represent individual patient results: * *p* < 0.05, ns *p* > 0.05; HBOT *n* = 14, Control *n* = 14.

No statistically significant differences in biochemical tests (CRP, D-Dimers, ferritin, LDH) were observed between the HBOT and C groups at baseline and follow-up (Table 2). At the end of the study, statistically significantly lower values of CRP [mg/dL]: W = 35, *p* = 0.042), ferritin [mg/mL]: W = 28, *p* = 0.045) and LDH [µ/L]: W = 28, *p* = 0.045 were observed vs. baseline in the HBOT group, but no difference was observed for D-Dimers [mg/mL]: *p* = 0.27) and procalcitonin (PCT [mg/mL]): *p* = 0.108) (Figure 4). In group C, none of the parameters reached statistical significance (CRP [mg/dL]: *p* > 0.999; ferritin [mg/mL]: *p* > 0.999; LDH [µ/L]: *p* > 0.999), D-Dimers [mg/mL]: *p* > 0.999; PCT [mg/mL]: *p* = 0.544).

### 3.5. Complete Blood Count (CBC)

**Figure 5:**
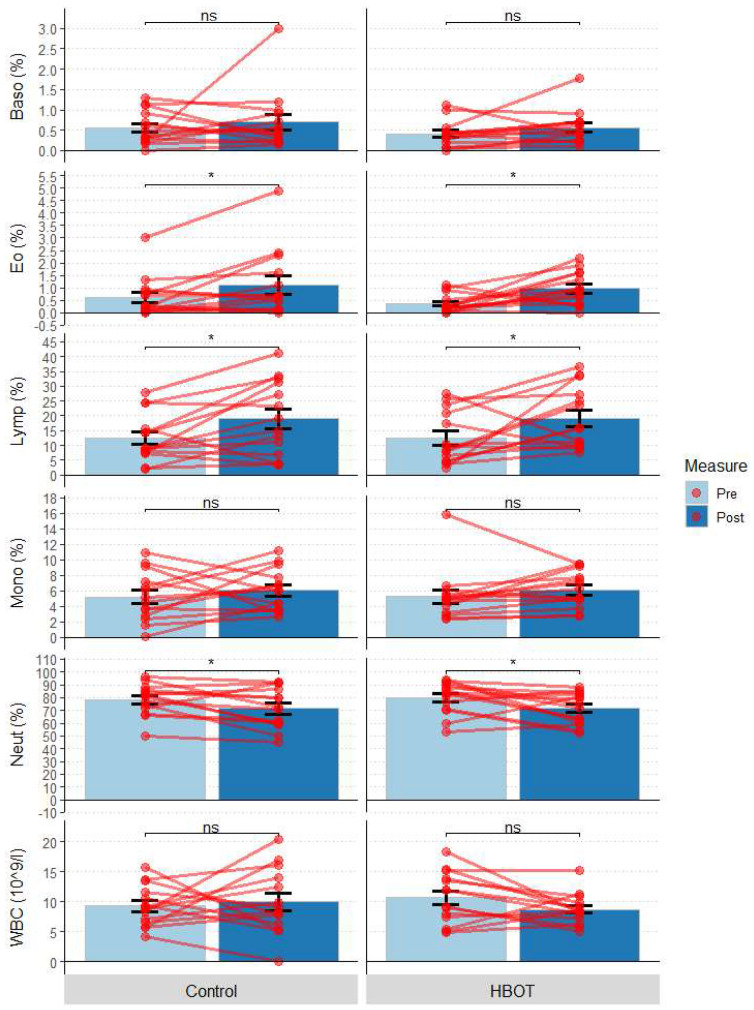
Change in leukocyte levels and leukocyte fraction at the first measurement (Pre) and 10 days after study initiation (Post); bars represent mean, error bars represent standard error, red lines represent individual patient results; * *p* < 0.05, ns *p* > 0.05; HBOT *n* = 14, Control *n* = 14.

No statistically significant CBC differences (White blood cells—WBC, lymphocytes—Lymp, neutrophils—Neut, monocytes—Mono, basophils—Baso, eosinophils—Eo) were observed between HBOT and C groups at baseline (*p* > 0.05)—Table 3. At the end of the study, statistically significant increase in eosinophilia (Eo, W = 0, *p* = 0.045), lymphocytes (Lymp, W = 13, *p* = 0.021) and statistically significant decreases in neutrophils (Neut, W = 27, *p* = 0.035) were observed in both groups (Figure 5). These changes were not statistically significantly different between the groups (*p* > 0.05). Other parameters (WBC, Mono, Baso) remained unchanged in both groups (*p* > 0.05).

### 3.6. Immunology

#### 3.6.1. T-Cell Subpopulations

**Figure 6:**
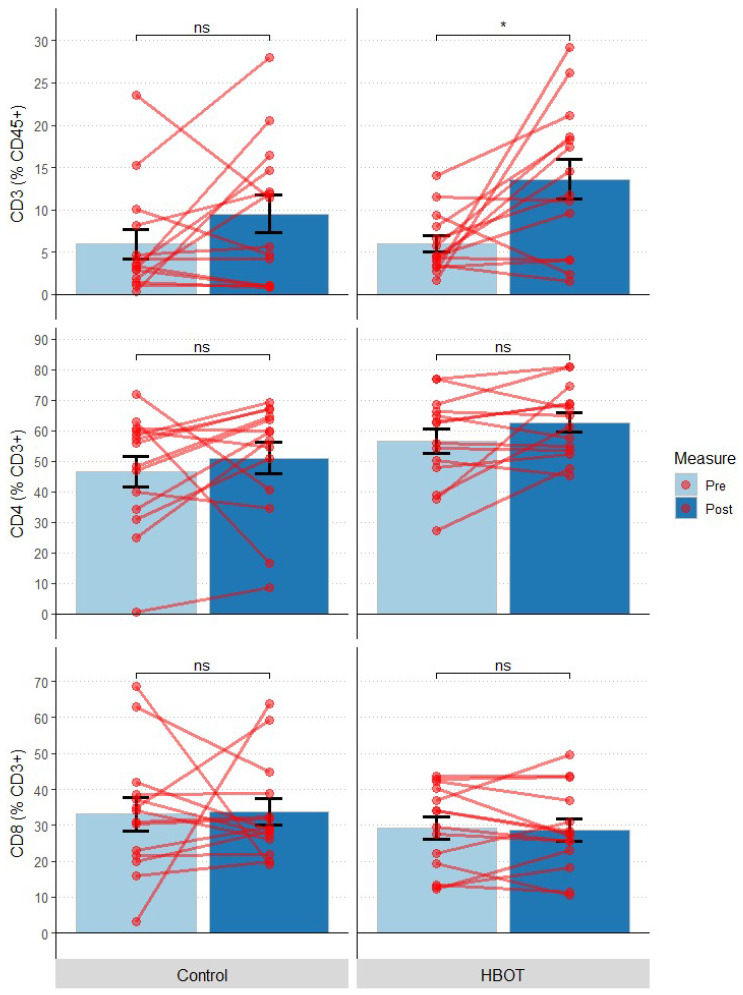
Change in the fraction of T-cells and their subpopulations; at first measurement (Pre) and 10 days after study onset (Post); bars represent mean, error bars represent standard error, red lines represent individual patient results; *—*p* < 0.05, ns—*p* > 0.05; HBOT *n* = 14, Control *n* = 14.

The following T-cell subpopulations were distinguished: CD3, CD4, CD8. There were no statistically significant differences in CD3 (% in CD45+), CD4 or CD8 lymphocyte percentages between HBOT and C groups at baseline (*p* > 0.05)—Table 4. At the end of the study, a statistically significant change was observed for CD3 (% within CD45+) in the HBOT group, where this value significantly doubled (CD3 (% in CD45+): F = 8.175, *p* = 0.042). Other parameters (CD4, CD8) remained unchanged in both groups (*p* > 0.05) (Figure 6).

#### 3.6.2. Interleukin 6

**Figure 7:**
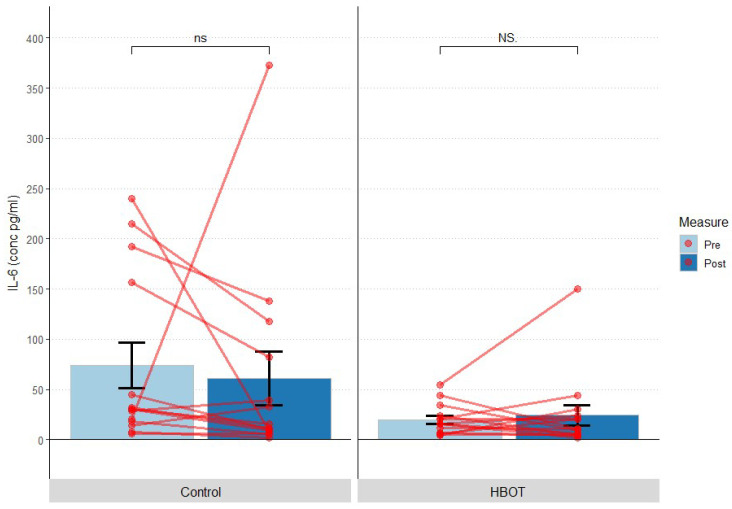
Change in interleukin 6 levels at first measurement (Pre) and 10 days after study onset (Post); bars represent mean, error bars represent standard error, red lines represent individual patient results; ns *p* > 0.05, NS *p* > 0.999; HBOT *n* = 14, Control *n* = 14.

Statistically significantly higher levels of interleukin 6 (IL-6) were observed in C group vs. HBOT group before the study (IL-6 (pg/mL): U = 142; *p* = 0.044, but not after study completion (IL-6 (pg/mL): U = 118; *p* = 0.74)—Table 5. No significant intra-group differences were shown in the “pre” measurement (baseline measurement) and “post” measurement (final measurement) (HBOT: *p* > 0.999; C: *p* = 0.161) (Figure 7). After exclusion of deceased subjects (all in the C group), IL-6 values in the control and HBOT groups did not differ significantly both at baseline (*p* = 0.298) and in the final measurement (*p* > 0.999).

## 4. Discussion

This study confirmed the feasibility and safety of HBOT in patients with moderate-to-severe SARS-CoV-2 pneumonia during the period of high infectiousness and requiring continuous delivery of high oxygen flows. No cases of life-threatening complications were observed in the HBOT group. After treatment of half of the planned population, persistence of significant mortality was observed only in the non-HBO group (21%, 3 of 14 patients), whereas no mortality was reported in the HBO group (0%, 0 of 14 patients) despite the same disease severity parameters in both groups at randomisation. This difference did not reach statistical significance but was close (*p* = 0.067) to such significance. With a small sample size, this strongly suggests beneficial effects of HBO on reduction of mortality.

Other reports describing the effectiveness of using HBOT in patients with COVID-19 were also published after the start of our study. In one, Guo et al. described two cases of severe SAR-Cov-2 pneumonia, which improved after HBOT [43]. Thibodeaux et al. published a paper evaluating the efficacy of HBOT in preventing artificial ventilation in patients with COVID-19 [44]. They used 1 to 6 sessions of HBO (2.0ATA for 90 min in a monoplace chamber) in 5 patients with tachypnoea and low SpO2 values despite oxygen therapy. They observed improvement in all patients and no need for artificial ventilation; inflammatory parameters declined. The authors concluded that “This small sample of patients exhibited dramatic improvement with HBOT. Most importantly, HBOT potentially prevented the need for mechanical ventilation. Larger studies are likely to define the role of HBOT in the treatment of this novel disease”. Boet et al. published a systematic review in September 2021 on the safety and efficacy of HBOT in patients with COVID-19 [45]. None of the 6 publications accepted for analysis included RCTs. The conclusions supported the safety of HBO in hypoxaemic patients with COVID-19 and indicated the need for RCTs. Oliaei et al. in 2021 published an independent systematic review [46] describing 8 papers accepted for analysis. In their summary, the authors wrote, “Overall, HBOT seems to be a safe and effective oxygenation method in patients with COVID-19. However, there is limited knowledge and evidence regarding the effects and mechanism of HBOT in COVID-19 treatment, and further evaluations require extensive well-designed studies.” Later on, newer report from prospective cohort study was published by Palaniappan T. et al. showing positive results of three hyperbaric sessions in monoplace chambers with partial pressure of oxygen between 1.50 to 1.80 ATA for approximately 45 min on pulse saturation [47].

The first RCT reporting the use of HBO as adjunctive treatment in patients with COVID-19 was published on 14 December 2021 by Cannellotto et al. [48]. For this multicentre study, 80 patients were planned to be recruited (20 to two treatment protocols each), but due to reaching the study objective, the study was discontinued after “interim analysis”. Ultimately, 40 patients were recruited in this study (20 in the control group and 20 in the HBO group). HBOT consisted of at least 5 sessions of 90 min of oxygen therapy at 1.45 ATA in a monoplace chamber. It was concluded that, compared to the control group, HBOT led to a more rapid reduction in the duration of hypoxaemia; no statistically significant effect was observed on ARDS severity, mechanical ventilation or 30-day mortality. This publication was also reported in an editorial in the journal [49]. Kjellberg et al. also published an RCT for HBOT in COVID-19 [50], but there are no published results from this study to date. Gorenstein et al. presented a comparison of 20 patients with COVID-19 treated prospectively with HBOT (90 min of stay under 2.0 ATA in a single-site chamber to a maximum of 5 sessions) with an appropriately matched control group in which HBOT was not used [51]. All patients had normobaric oxygen treatment at flow rates between 2 and 15 Lpm. Of the 20 patients treated with HBOT, only 2 (10%) required intubation and eventually died; the others were discharged from hospital in good condition. Of the 60 patients in the comparison group, 18 patients (30%) were intubated, 13 (22%) died and 3 (5%) required continued hospitalisation. Assuming no further deaths in the control group, the analysis showed a statistically lower rate of requiring artificial ventilation (*p* = 0.046), but no significance for the difference in mortality (*p* = 0.14). As in other similar work, the final conclusion was “Though limited by its study design, our results demonstrate the safety of hyperbaric oxygen among COVID-19 patients and strongly suggests the need for a well-designed, multi-centre randomized control trial.”

In our study, we decided to focus on the biochemical, cellular and cytokine effects of HBOT in COVID-19 patients. At the time of preparation of the study protocol, there was no published data on immunological aspects of HBOT in COVI-19. While we are still awaiting some other studies to be reported, the only other research on HBOT efficacy as an adjuvant for the Systematic Inflammation Reduction in patients with SARS-CoV-2 infection was published by the Mexican group [52]. They compared 36 patients effectively randomized to HBOT group (with 10 daily sessions of 2.0 ATA oxygen for 130 min with air breaks in a multiplace chamber) with 42 patients in the control group. They observed significant decrease of D-Dimer, ferritin, DHL, CRP and ESR values to normal or reference values in the HBOT group, while such decrease was not so significant in the control group.

No statistically significant differences were observed between groups C and HBOT in the baseline values of the parameters tested (except for IL-6, see further below), demonstrating the effectiveness of randomisation.

When concerning IL-6, a statistically significant difference in Interleukin 6 (IL-6) levels was observed between groups C and HBOT at baseline, while no statistically significant change was observed in either group. IL-6 levels were 2-fold higher in the control group. High levels of IL-6 were observed in patients who died (all in the control group). After performing an additional analysis that did not include deceased patients, no statistically significant differences were found between groups or time points.

In both C and HBOT groups, the pre-set oxygenation parameters based on SpO2 and PaO2/FiO2 were maintained, which proves treatment efficacy. In the HBOT group, this was achieved by using lower passive oxygen therapy flows. However, the statistical difference in the amount of oxygen used between groups C and HBOT does not translate into therapeutic efficacy. In fact, it is well known fact for all clinician that using SpO2 or PaO2/FiO2 index in non-intubated patients is prone to even slight fluctuations in oxygen delivery, due to patient movement, speaking or drinking.

A statistically significant increase in the percentage of eosinophilia and lymphocytes and a decrease in neutrophils were observed in both groups during the study. These changes did not differ between C and HBOT groups.

During the study, statistically significant changes only in the HBOT group included decreases in CRP, ferritin and LDH and increases in CD3. This confirms that indeed HBOT influences at least some biochemical and cellular parameters of COVID-19 patients.

The most interesting, and not yet reported fact is a change in CD3+ percentage observed only in HBOT group, and not in C group. Lower CD3+ percentage has been shown as a predictor of COVID-19 severity [53]. In our study, patients undergoing HBOT treatment were characterized by increase in T cell contribution during 10-day observation, while in the control group the CD3+ percentage remained low, as in other COVID-19 studies [54]. Restoration of CD3+ levels can therefore suggest beneficial effect of HBOT on modulation of immune responses. As simultaneous decrease of CRP could be observed, CD3+ induction by HBOT did not indicate inflammation. In fact, hyperbaric oxygen was shown to have immunosuppressive properties, e.g., during autoimmune diseases [55]. On the other hand, HBOT-induced immunosuppression was suggested to compromise immunity in a tumour model [56]. In our study, the disease was not exacerbated, as measured by NEWS. Our results suggest that immunomodulatory effect of HBOT consists of alleviation of inflammation and partial restoration of disturbed T cell responses; however, the exact mechanism could not be explained. Possible molecular and cellular mechanisms mediating beneficial effect of HBOT were recently reviewed [19]. They consist of, but only, changes in markers of oxi-dative stress, inflammation, or angiogenesis, which can probably explain the effects we observed in our study. As documented in many other clinical situations, HBO reduces the inflammatory process by reversing local hypoxia [39]. Induction of oxygen free radicals and fluctuations in hypoxia-inducible factor-1 (HIF-1) levels reduce neutrophil activation by altering beta-2 integrin function and modifying the release of heme oxygenase 1 (HO-1) [20]. HIF-1 induces transcription factors regulating inflammation, mainly Nrf2 and NFkB, which has longer-lasting effects than hyperbaric oxygen respiration alone [21]. Similar long-term effects are observed for HBOT-induced stimulation of bone marrow stem cells [23]. In baseline studies, we also observed a decrease in pro-inflammatory cytokines, mainly IL-1β, IL-6, and TNF-α, and an increase in anti-inflammatory interleukins, mainly IL-4, IL-10, and IL-13 [22,57]. HBO also inhibited the production of pro-inflammatory cytokines by blood-borne macrophages (monocytes) induced by external stressors [58].

Additionally, though limited by its study design, our results demonstrate the safety of hyperbaric oxygen among COVID-19 patients and strongly suggests the need for a well-designed, multi-centre randomized controlled trial.

A relatively up-to-date inventory of registered scientific studies has been published by UHMS (UHM 2020). Also published are the positions of the major scientific organisations involved in hyperbaric medicine on the use of HBOT in patients with COVID-19: EUBS/ECHM [59] and UHMS [60].

The next scientific question could be related with the possible use of HBOT in so-called “long COVID-19” [61]. We are waiting for the upcoming prospective studies [62].

The present study also demonstrates the feasibility of successful and safe HBO sessions in patients with moderate-to-severe SARS-CoV-2 pneumonia during a period of high infectiousness and requiring continuous supply of high oxygen flows. The issue of safety of the staff caring for patients on high oxygen flows or in the confined space of a hyperbaric chamber has also been raised in other publications [63,64,65,66,67,68].

We acknowledge the limitations of our study. One is that the small sample size restricts the study results. Still, it answers the primary endpoint on HBOT safety in COVID-19 patients and on the secondary endpoints related to affecting the immunological status of COVID-19 patients by the hyperbaric oxygenation. However, it is not powered enough to either confirm or deny the clinical efficacy of HBOT in the general population of patients with COVID-19. Another limitation is that the treating physicians determined standard care for COVID-19 in both groups; thus, it is possible that differences in standard care throughout the trial enrolment period could influence outcomes.

## 5. Conclusions

This analysis of our prospective RCT on using HBOT in severe COVID-19 confirmed the effectiveness of randomisation (except for IL-6), showed a clear trend towards fewer deaths in the HBOT group, confirmed the possibility of safely conducting HBOT in this patient group, showed a lower need for normobaric oxygenation in the group of patients treated with HBOT and demonstrated significant beneficial effects of HBOT on CRP, ferritin, LDH and CD3.

Considering the obtained results, including the positive response to the question regarding the effect of HBOT on the inflammatory response, the declining rate of patient recruitment, the changing phenotype of the SARS-CoV-2 virus and the changing immune status of the patients due to COVID-19 or subsequent doses of vaccination, it was concluded that continuing the study according to the previous protocol has little chance of increasing the strength of validity of the results achieved. It was therefore decided that this study should be terminated.

## Figures and Tables

**Table 1:** Statistics for blood gas results at baseline and day 5.

	Group	Pre			Post		
		M	SE	IQR	M	SE	IQR
Oxygen supply (L/min)	HBOT	11.07	2.15	10.50	4.79	0.84	3.75
	Control	12.50	3.44	5.75	9.62	2.99	10.00
PiO2/FiO2	HBOT	171.52	31.60	76.96	214.88	31.27	143.78
	Control	129.34	27.68	36.55	134.91	24.21	132.35
SpO2 (%)	HBOT	96.26	0.88	5.05	93.19	1.84	5.15
	Control	93.30	1.96	5.55	92.68	1.94	5.60

M—mean; SE—standard error; IQR—interquartile range.

**Table 2:** Biochemical tests at the beginning and at the end of the study in HBOT and control group.

	Group	Pre			Post		
		M	SE	IQR	M	SE	IQR
CRP (mg/dL)	HBOT	3.83	1.16	3.65	1.54	0.77	0.75
	Control	4.46	0.93	4.55	4.54	1.76	5.50
D-Dimer (mg/mL)	HBOT	1.29	0.29	0.60	1.37	0.32	0.51
	Control	4.92	2.86	1.13	5.46	2.90	2.34
Ferritin (mg/mL)	HBOT	1186.36	355.55	749.25	741.63	126.43	695.00
	Control	745.31	198.81	1034.00	580.68	154.46	736.70
LDH (µ/L)	HBOT	304.53	40.39	147.75	258.93	39.55	130.25
	Control	267.12	62.63	328.29	246.40	64.01	324.30
PCT (mg/mL)	HBOT	0.086	0.018	0.055	0.071	0.014	0.025
	Control	0.174	0.056	0.092	0.154	0.056	0.062

CRP—C-reactive protein; LDH—lactate dehydrogenase; PCT—procalcitonin, M—mean; SE—standard error; IQR—interquartile range.

**Table 3:** CBC for input and output results.

	Group	Pre			Post		
		M	SE	IQR	M	SE	IQR
WBC (10^9^/L)	HBOT	10.65	1.14	6.13	9.59	0.85	3.54
	Control	9.28	0.90	4.62	8.05	0.97	3.64
Lymp (%)	HBOT	12.41	2.36	14.77	19.69	3.05	17.55
	Control	12.45	2.17	7.56	19.40	3.26	21.40
Neut (%)	HBOT	79.94	3.34	16.68	71.41	3.72	22.85
	Control	78.44	3.23	12.27	70.76	4.14	24.93
Mono (%)	HBOT	5.27	0.88	2.12	5.64	0.61	2.93
	Control	5.22	0.86	4.12	6.28	0.78	4.46
Baso (%)	HBOT	0.41	0.09	0.23	0.50	0.08	0.43
	Control	0.56	0.11	0.59	0.51	0.09	0.44
Eo (%)	HBOT	0.37	0.10	0.34	0.90	0.18	0.93
	Control	0.62	0.21	0.73	1.14	0.36	1.32

WBC—white blood cells, Lymp—lymphocytes, Neut—neutrophils, Mono—monocytes, Baso—basophils, Eo—eosinophils, M—mean; SE—standard error; IQR—interquartile range.

**Table 4:** Immunology for input and output results.

	Group	Pre			Post		
		M	SE	IQR	M	SE	IQR
CD3 (% CD45+)	HBOT	5.97	0.95	4.05	13.76	2.29	11.03
	Control	5.96	1.73	5.18	9.58	2.23	11.65
CD4 (% CD3+)	HBOT	56.41	3.98	17.45	62.80	3.15	14.80
	Control	46.58	5.05	24.00	52.19	5.42	23.18
CD8 (% CD3+)	HBOT	29.38	3.11	19.33	28.78	3.08	11.63
	Control	33.10	4.62	16.23	32.86	3.87	14.05

M—mean; SE—standard error; IQR—interquartile range.

**Table 5:** IL-6 levels at treatment onset and at the final measurement.

	Group	Pre			Post		
		M	SE	IQR	M	SE	IQR
IL-6 (pg/mL)	HBOT	14.80	19.91	4.02	17.35	24.47	10.18
	Control	110.15	74.10	22.89	62.76	60.79	26.77

IL-6—interleukin 6, M—mean; SE—standard error; IQR—interquartile range.

## Data Availability

All data are available from the corresponding author upon reasonable request.
